# Sustainable Production of Biosurfactant from Agro-Industrial Oil Wastes by *Bacillus subtilis* and Its Potential Application as Antioxidant and ACE Inhibitor

**DOI:** 10.3390/ijms231810824

**Published:** 2022-09-16

**Authors:** Dominika Ciurko, Żaneta Czyżnikowska, Anna Kancelista, Wojciech Łaba, Tomasz Janek

**Affiliations:** 1 Department of Biotechnology and Food Microbiology, Wrocław University of Environmental and Life Sciences, 51-630 Wrocław, Poland; 2 Department of Inorganic Chemistry, Faculty of Pharmacy, Wroclaw Medical University, 50-556 Wrocław, Poland

**Keywords:** *Bacillus subtilis*, biosurfactant, bioactive lipopeptide, antioxidant, ACE inhibitors

## Abstract

The microbial conversion of agro-industrial oil wastes into biosurfactants shows promise as a biomass refinery approach. In this study, *Bacillus subtilis* #309 was applied to produce surfactin using rapeseed and sunflower cakes, the most common oil processing side products in Europe. Studies of the chemical composition of the substrates were performed, to determine the feasibility of oil cakes for surfactin production. Initially, screening of proteolytic and lipolytic activity was performed to establish the capability of *B. subtilis* #309 for substrate utilization and hence effective surfactin production. *B. subtilis* #309 showed both proteolytic and lipolytic activity. The process of surfactin production was carefully analyzed by measurement of the surfactin concentration, pH, surface tension (ST) and emulsification index (E_24_). The maximal surfactin concentration in the sunflower and rapeseed cake medium reached 1.19 ± 0.03 and 1.45 ± 0.09 g/L, respectively. At the same time, a progressive decrease in the surface tension and increase in emulsification activity were observed. The results confirmed the occurrence of various surfactin homologues, while the surfactin C_15_ was the dominant one. Finally, the analysis of surfactin biological function exhibited antioxidant activity and significant angiotensin-converting enzyme (ACE)-inhibitory activity. The half-maximal inhibitory concentration (IC_50_) value for ACE inhibition was found to be 0.62 mg/mL for surfactin. Molecular docking of the surfactin molecule to the ACE domains confirmed its inhibitory activity against ACE. Several interactions, such as hydrophobic terms, hydrogen bonds and van der Waals interactions, were involved in the complex stabilization. To the best of our knowledge, this is the first report describing the effect of a lipopeptide biosurfactant, surfactin, produced by *B. subtilis* for multifunctional properties in vitro, namely the ACE-inhibitory activity and the antioxidant properties, using different assays, such as 2,2-azinobis (3-ethyl-benzothiazoline-6-sulfonic acid (ABTS), 2,2-diphenyl-1-picrylhydrazyl (DPPH) and ferric reducing antioxidant power (FRAP). Thus, the ACE-inhibitory lipopeptide biosurfactant shows promise to be used as a natural antihypertensive agent.

## 1. Introduction

Surfactants are chemical compounds widely used in industry, due to the ability to reduce surface tension between two phases. A surfactant molecule is built of two functional parts: a polar, hydrophilic head and a tail that is nonpolar and lipophilic. However, surfactants are manufactured with petroleum or derivatives, which contributes to their toxicity [1,2]. The cycle of toxicity includes synthesis, disposal and subsequent, not entirely controlled release to the environment [2]. In the first instance, surfactants released into the environment affect algae and other microorganisms through increased cell membrane permeability and the subsequent disintegration of the cell structure. When the concentration is high enough, a harmful effect is observed in relation to fish, which absorb chemicals through their body surface, and to animals as well as humans because of meat consumption. Surfactants affect enzyme activity, causing a variety of severe afflictions [3]. Nowadays, due to the high attention to health and increasing environmental awareness, the growing popularity of biosurfactants can be observed. 

Biosurfactants possess similar characteristic properties to synthetic surfactants, but are synthesized by living cells; hence, they are environmentally friendly. In addition, biosurfactants are described as compounds easily disintegrated by microorganisms, without posing any ecological harm. They are characterized by low toxicity, high selectivity and a low critical micellar concentration (CMC). Biosurfactants remain active under extreme temperatures, pH and salinity, and can be produced by yeast, filamentous fungi and bacteria from various substrates [4,5]. Classified as secondary metabolites, biosurfactants are produced when the culture reaches a certain physiological state and cell density, under nutrient availability. Biosurfactant synthesis involves various metabolic pathways and normally occurs in the stationary growth phase. However, it may also proceed under optimal growth conditions. Microbial synthesis of the hydrophilic and hydrophobic fractions occurs with the use of the same substrate, but through various biosynthetic pathways [6]. 

Biosurfactants possess a wide spectrum of medical applications. They can act as antibiotic, antiviral and antifungal agents. Cyclic lipopeptides such as surfactin, fengycin, iturin, bacillomycin and mycosubtilin produced by *B. subtilis*; lichenysin, pumilacidin and polymyxin B secreted, respectively, by *Bacillus licheniformis*, *Bacillus pumilus* and *Bacillus polymyxa*; daptomycin from *Streptomyces roseosporus*; and viscosin from *Pseudomonas* are the most widely reported classes of biosurfactants with antimicrobial activity [7]. 

Moreover, these compounds show antitumor activity and immunomodulatory properties [8]. Biosurfactants also can act as inhibitors of ACE, and therefore they can be applied as antihypertensive agents [9]. 

The most common classification of biosurfactants is based on the chemical nature. Biosurfactants are classified into glycolipids, lipopeptides, lipoproteins, phospholipids, fatty acids (FAs), polymeric surfactants and particulate surfactants. Surfactin, one of the most active biosurfactants found in nature, is a bacterial cyclic lipopeptide produced by the genus *Bacillus* [5,10]. *B. subtilis* is the most common producer of surfactin but it can also be found in cultures of *B. pumilus*, *B. licheniformis* and *Bacillus amyloliquefaciens*. The molecule of surfactin is composed of a heptapeptide (ELLVDLL) interlinked with a β-hydroxy fatty acid (FA). The FA chain adopts the form of a cyclic lactone ring and is composed of several carbon atoms ranging from 12 to 16. The molecule contains two negative charges thorough glutamyl and aspartyl residues in the structure of the peptide chain. Usually, several isoforms of surfactin, as a mixture of peptidic variants with a different aliphatic chain length, are secreted [11]. Surfactin represents an exceptionally wide spectrum of activity, which makes it an appropriate compound for medical applications. The antibacterial, antifungal, antiviral and anti-mycoplasmatic effects are only few among the wide range of properties. Surfactin is known to strongly interact with the process of biofilm formation. It prevents bacterial adhesion and decreases the potential for nosocomial *Salmonella typhimurium*, *Salmonella enterica*, *Escherichia coli* and *Proteus mirabilis* infections. In addition, surfactin exhibits strong anti-inflammatory, antitumor and thrombolytic activity [12]. In addition to the properties described above, surfactin has also been related to the inhibition of fibrin clot formation, the induction of ion channel formation in lipid bilayer membranes, the inhibition of cyclic adenosine monophosphate and the inhibition of platelet and spleen cytosolic phospholipase A2 (PLA2) [8]. 

The type of biosurfactant is strongly influenced by both the substrate and the production microorganism. As reported by Ribeiro et al. [6], the application of alternative carbon sources leads to the production of different chemical structures. In this time of ecological crisis, various industrial by-products are being investigated as potential substrates for biosurfactant production. 

As described by Banat et al. [13], biosurfactants can be produced using agro-industrial wastes such as beet or sugarcane molasses, straw of wheat/rice, cassava flour wastewater and hulls of soy/corn. In addition, animal fats and oil-processing wastes are extensively examined as substrates. There is growing interest in coconut, peanut or soybean cake, oil mill wastewater and canola meal. Biosurfactant production can be carried out with the participation of various residues from the coffee (coffee pulp or husks), fruit (banana waste, fruit pomace, carrot waste) and food (frying edible oils and fats, potato peels) industries.

The purpose of the present study was to produce a lipopeptide biosurfactant from *B. subtilis* #309 using low-cost agro-food wastes. Surfactin production was performed using rapeseed and sunflower cake, the most common oil processing side products in Europe, to maintain the cost-effectivity of the entire process. In order to confirm the suitability of the conditions used for surfactin production, detailed analysis of the substrate and the screening of the proteolytic and lipolytic activity of *B. subtilis* #309 was performed. Moreover, the surfactin was studied as a new potential antioxidant and ACE-inhibitory active substance. Finally, the molecular docking of surfactin was performed to confirm the possibility of surfactin’s application as an effective ACE inhibitor.

## 2. Results and Discussion

### 2.1. Sunflower and Rapeseed Cake Composition 

Sunflower and rapeseed cakes used in this study were characterized by high dry matter content, 93.8% and 93.1%, respectively (Table 1). However, there was a strong variation in the crude protein concentration, reaching 21.4% and 30.2% (in dry matter), respectively, for sunflower and rapeseed cake. Likewise, significant differences in the FA profiles were detected. Sunflower and rapeseed cake were significantly different in terms of oleic and linoleic acid concentrations. The content of oleic acid reached 34.96% and 77.84%, while the linoleic acid concentration totaled 55.67% and 8.05%, respectively, for sunflower and rapeseed cake. Analysis showed the presence of palmitic acid, as a third important fraction in the FA profiles of sunflower and rapeseed cake.

The chemical composition of oil seed cake depends on numerous extrinsic factors, such as genetic traits, cultivation climate, soil and oil production process conditions [14], and hence some strong variations are often observed. According to Gültekin Subaşı et al. [15], the protein and fiber concentrations in sunflower cake vary in the range of 29.0–61.06% and 4.30–45.00%, respectively, while, according to Geneau-Sbartaï et al. [14], the protein content varies between 29.4% and 35.0%.

The estimated FA composition of sunflower cake was in line with the results of research performed with the aid of sunflower oil. Studies of Kostik et al. [16] showed linoleic acid (59.5%) as the dominant fraction, followed by oleic (31.5%) and palmitic (3.7%) acid. Chowdhury et al. [17] further confirmed the presence of linoleic (46.02%), oleic (45.39%) and palmitic (6.52%) acid. In the studies of Leming and Lember [18], the estimated protein concentration of rapeseed cake (30.6%) was almost identical to the result obtained in our research. A comparable result was found in the study of Jeong et al. [19], where the protein concentration reached 31.58%. 

The content of oleic acid in rapeseed cake reported in our research was high, compared to the results of rapeseed oil analysis performed by other authors. According to the literature sources, the oleic acid concentration of rapeseed oil totaled 59.5% [16], 61% [20] or 62.5% [21]. Significant differences were also noted in the content of linoleic and palmitic acid. In research involving rapeseed oil, the linoleic acid concentration was estimated at 18.8% [16], 21% [20] or 19.6% [21], and palmitic acid at 5.2% [16], 7% [20] and 4.3% [21].

Due to the high protein concentration and the presence of saturated and unsaturated FA, both sunflower and rapeseed cakes are by-products with great application potential for biosurfactant production, providing the essential chemical constituents. In addition, with reference to the theory of hydrophilic and hydrophobic inducers of biosurfactant production, the FAs of sunflower and rapeseed cakes serve as a secondary carbon source for microbial growth and assist the biosynthesis of the FA moieties of biosurfactant molecules [22]. Moreover, due to the significant variation in the FA profile of the sunflower and rapeseed cake, a study of the FAs’ effects on the production of surfactin homologues can be performed.

### 2.2. Kinetics of Surfactin Production in B. subtilis #309 Culture on Oil Cakes 

**Figure 1:**
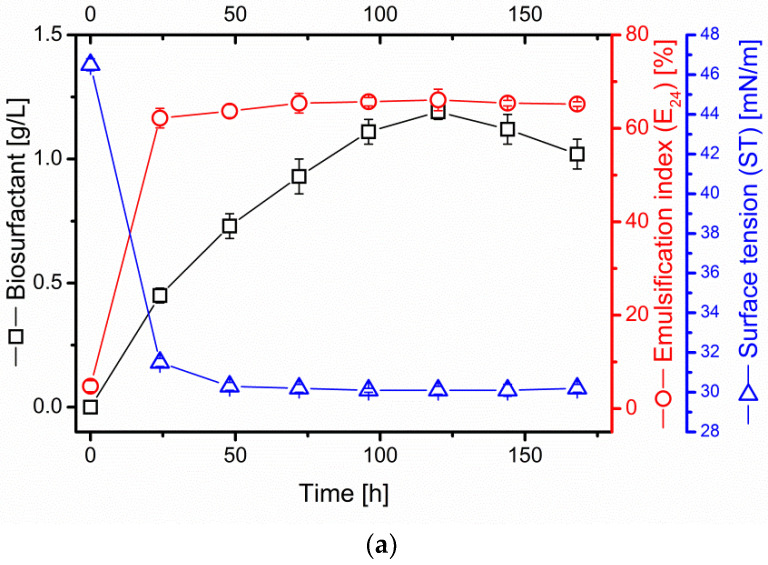

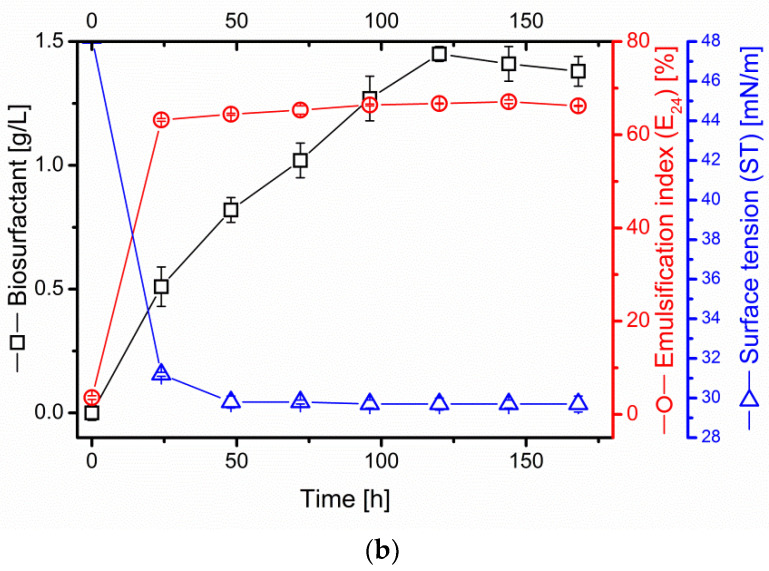
Effect of (**a**) sunflower and (**b**) rapeseed oil cake substrates (5% *w*/*v*) in shake-flask cultures on biosurfactant production, pH, emulsification index (E_24_) and surface tension (ST). The cultures were performed at 37 °C and 160 rpm for 168 h. The results represent the mean ± standard deviation of triplicate assays.

The surfactin production process in the cultures of *B. subtilis* #309 in the sunflower and rapeseed cake medium displayed a similar course. After 24 h, a significant drop in the ST from the initial 46.5 ± 0.3 mN/m to 31.5 ± 0.2 mN/m in the sunflower cake medium and from 48.2 ± 0.3 mN/m to 31.2 ± 0.1 mN/m in the rapeseed cake medium was observed (Figure 1a,b).

Till the end of the biosynthesis process, the ST remained low and stable, reaching minimal values of 30.1 ± 0.1 mN/m and 29.7 ± 0.2 mN/m, respectively. In addition, the emulsification index (E_24_ (%)) increased rapidly after 24 h of *B. subtilis* #309 cultivation, reaching 62.2 ± 2.1% in the sunflower and 63.2 ± 0.3% in the rapeseed cake medium. The maximum value of E_24_ (%) was estimated at 66.1 ± 0.3% and 67.1 ± 0.4%, respectively (Figure 1a,b). A logarithmic increase in the surfactin content was observed. The maximum concentration was detected at 120 h of the culture. In the sunflower cake medium, surfactin production reached 1.19 ± 0.03 g/L, while, in the rapeseed cake medium, the surfactin concentration was nearly 20% higher, quantified at 1.45 ± 0.09 g/L (Figure 1a,b). Surfactin biosynthesis was additionally monitored using the pH value. However, no significant variations were observed. Slight alkalinization of the culture medium occurred, from the initial 6.29 ± 0.23 in the sunflower and 6.03 ± 0.14 in the rapeseed cake medium to 8.91 ± 0.12 and 8.98 ± 0.09, respectively. 

Several by-products have been previously studied as substrates for biosurfactant production. Among them, waste automobile oil was applied in cultures of native and chemically mutated strains of *B. subtilis*. The emulsification index (E_24_) has been used to determine the presence of microbial surfactant. In the culture of the native strain, the emulsification index reached 42%, while the best result, obtained for the mutant *B. subtilis* MS1, totaled 50.53% [23]. 

In other studies, unconventional substrates, such as waste glycerol, cheese whey, clarified cashew apple juice and sunflower oil, were used for biosurfactant production by *B. subtilis* ICA56. Biosurfactant production by this strain, previously isolated from a Brazilian mangrove soil, occurred with the best efficiency in the waste glycerol medium. In this condition, the produced biosurfactants promoted a significant reduction in the ST (28 mN/m) and the formation of a stable emulsion in motor oil (92%). However, a significant ST reduction (36 mN/m) and relevant emulsification activity (90%) were detected in addition for the sunflower medium broth [24]. 

Waste frying oils were the subject of interest of Vedaraman and Venkatesh [25]. However, research performed using *B. subtilis* MTCC 2423 as a surfactin producer demonstrated lower usefulness of frying sunflower oil (WFSO) and rice bran oil (WFRBO) compared to oil cakes as substrates. The yield of surfactin production was lower, compared to the studies described in this paper. The surfactin production, after 120 h of bacterial cultivation, reached approximately 0.7 g/L (WFSO) and 0.45 g/L (WFRBO). At the same time, a similar ST reduction, from the initial, approximate value of 60 mN/m to the final value of 31.9 mN/m (WFSO) and 34.5 mN/m, was observed (WFRBO). As in our studies, emulsification of the culture broth, facilitating the microbial access to the substrates, was detected [25].

Solids of waste frying oil were applied by Oliveira and Garcia-Cruz (2013) for biosurfactant production using *B. pumilus* CCT 2487. The best result of surface tension reduction was 45 mN/m. At the same time, the cell-free broth did not form an emulsion with toluene [26]. In another study, olive oil mill waste (OMW), a common residue in the Mediterranean area, obtained after the extraction of olive oil, was applied at three concentrations (2%, 5% and 10%) as a carbon source for surfactin production [27]. However, in the culture of *B. subtilis* N1, a negative impact of the increasing substrate concentration on the surface tension was observed, resulting in its increase from 39.3 mN/m to 46.6 mN/m. Similar effects were noticed in relation to the lipopeptide production yield. Maximum production (3.12 mg/L) was detected in the culture with the lowest tested substrate concentration (2%). The inhibitory effect of olive oil mill waste for surfactin production was attributed to the abundance of phenolics found in OMW [27]. 

One more example of the use of oil wastes for biosurfactant production is the work of Abas et al. [28], where the treated palm oil mill effluent (POME) was applied. POME, applied at different concentrations as a carbon source for surfactin production, was efficiently utilized by *B. subtilis* ATCC 21332. The maximal surfactin concentration, quantified in the media containing 50% of POME, was 30–35 mg/L. The results of our study, as well as literature examples, support the suitability of sunflower and rapeseed cakes as substrates for the surfactin production process. We observed a substantial reduction in the ST, a significant increase in the emulsification index and the efficient production of surfactin, at a low production cost. Against the background of the cited examples, the production of surfactin using sunflower and rapeseed cakes is highly efficient. Moreover, such a high yield points to the lack of an inhibitory effect of oil cake compounds on bacterial metabolism.

### 2.3. The Profile of Proteolytic and Lipolytic Enzymes Involved in Oil Cake Decomposition

**Figure 2:**
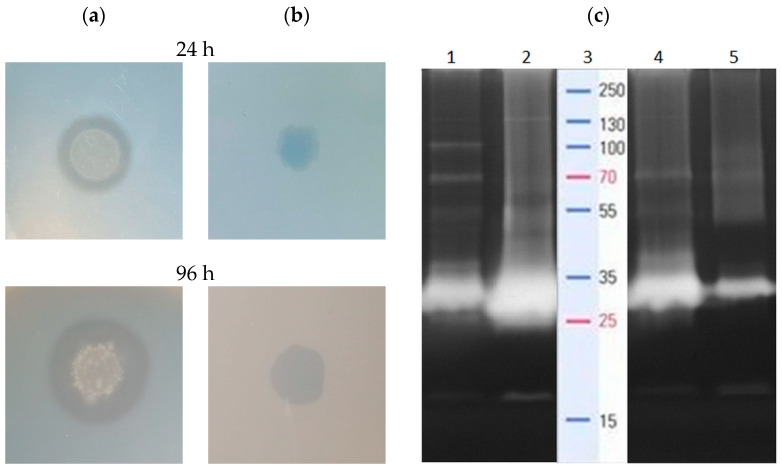
Proteolytic (**a**) and lipolytic (**b**) activity of *B. subtilis* #309. (**c**) Gelatin zymogram shows clear proteolytic bands of protein cleavage on 10% sodium dodecyl sulfate-polyacrylamide gel electrophoresis (SDS-PAGE) in the hours following *B. subtilis* culture conducted in the rapeseed and sunflower cake medium. Lane 1: rapeseed cake (24 h), lane 2: rapeseed cake (96 h), lane 3: molecular mass marker, lane 4: sunflower cake (24 h), lane 5: sunflower cake (96 h).

Initial screening of the proteolytic and lipolytic activity of *B. subtilis* #309, performed on a skim milk agar plate and tributyrin plate, confirmed the ability of this strain to secrete proteases and lipases. We observed clear hydrolysis zones, surrounding bacterial colonies, both after 24 and 96 h of incubation (Figure 2a,b). Expansion of the hydrolysis zones, indicating the progressive hydrolysis of the substrates, was observed over time.

*B. subtilis* #309 protease secretion was strongly induced by the presence of rapeseed cake protein in the culture medium, as determined with zymographic analysis. Although no proteolytic bands were observed after medium inoculation, significant activity was detected in the following hours. A wide spectrum of proteolytic enzymes with relative molecular masses of 130, 100, 70, 50, 40, 28 and 16 kDa were secreted after 24 h of bacterial cultivation (Figure 2c, lane 1). However, in the 96th hour, the intensity of the bands corresponding to the enzymes with molecular weights of 130, 28 and 16 kDa increased markedly, while the visibility of bands representing enzymes with molecular masses of 100, 70 and 55 kDa was clearly reduced (Figure 2c, lane 2). This pointed to the involvement of various proteolytic enzymes at different stages of rapeseed cake utilization. Likewise, several enzymes with molecular weights of 130, 70, 50, 40, 28 and 16 kDa were detected at the early stage of sunflower cake protein decomposition (24 h) (Figure 2c, lane 4). However, the gradual disappearance of the proteolysis band was observed as the process progressed. After 96 h of protein hydrolysis, a single proteolytic band of 28 kDa remained, as evidenced on the zymogram (Figure 2c, lane 5).

The profile of *B. subtilis* PF1 proteolytic enzymes, associated with the biosurfactant production process, was examined in the work of Bhange et al. [29]. In a medium composed of feather meal, potato peel and rapeseed cake, optimized for proteolytic and amylolytic activity and to maximize biosurfactant yield, the presence of four proteases, ranging from 97.4 to 45 kDa, was confirmed. At the same time, biosurfactant production, described with the emulsification index, reached approximately 40%. The performed research evidenced the production of numerous proteolytic enzymes involved in sunflower and rapeseed cake protein disintegration. The significant activity of the hydrolytic enzymes ensured the accessibility of building compounds essential for surfactin biosynthesis process.

### 2.4. Surfactin as an Oil Recovery Agent

Surfactin, due to its excellent surface activity, can readily release trapped oil and significantly improve oil recovery. In addition, its considerable emulsification activity facilities the emulsification of oil released into the mobile phase. These two properties meet the requirements for microbially enhanced oil recovery (MEOR). This points to surfactin as an agent of great potential for application in ex situ processes, supporting oil extraction from reservoirs and oil recovery from contaminated soils [30].

The oil removal efficiency of cell-free supernatants of *B. subtilis* #309 increased gradually with the surfactin concentration. Maximum rates of crude oil recovery, obtained at 120 h of culture, were 31.6% in sunflower and 37.1% in rapeseed cake supernatant (Table 2), in which the respective surfactin concentrations were 1.19 and 1.45 g/L (Figure 1).

In this study, the percentages of oil recovery were analyzed for the unpurified surfactin, due the high cost of the purification process. The intention was to develop a simple and cost-effective procedure, with prospective environmental application. Therefore, the application of cell-free fermentation media, containing significant amounts of highly active biosurfactant, produced on low-cost agri-food residues, fits this concept.

A similar approach was presented in the research of Bezza and Chirwa [31], where the cell free-supernatant containing a lipopeptide biosurfactant, produced in the culture of *B. subtilis* CN2 as the glycerol minimal salt medium (MSM), was investigated. The biosurfactant allowed the removal of 84.6 ± 7.1% of motor oil from contaminated sands. In another work, the ability of *B. atrophaeus* 5-2a (KP314029) to produce biosurfactants was evaluated using two media: BB containing beef extract, peptone and brown sugar, and BU using brown sugar and urea as the carbon and nitrogen sources. The efficiency of the cell-free fermentation broths for crude oil removal was significant, reaching 94.0 ± 0.092% (BB) and 90.0 ± 0.057% (BU) [32].

Another approach was presented in the research of Liu et al. [33], where two standard media, glucose and Luria–Bertani (LB) medium, were applied for surfactin production using modified *B. subtilis* BS-37. At the concentration of 300 mg/L purified surfactin, produced on LB and glucose media, the authors could recover 96.8 ± 1.0% and 96.7 ± 1.4% of oil from sand, respectively. However, the use of a genetically modified strain, standard laboratory medium and purified biosurfactant has some drawbacks. In addition to the increased cost of this process, genetic modification and biosurfactant purification processes are time- and work-consuming procedures.

All the aspects of the selected production methodology should be considered before process development. The implementation of genetic engineering and synthetic culture media may positively impact the final oil recovery, but, at the same time, they render the entire procedure unsuitable for large-scale environmental applications.

### 2.5. The Profile of Surfactin Homologues in the Sunflower and Rapeseed Cake Media 

**Figure 3:**
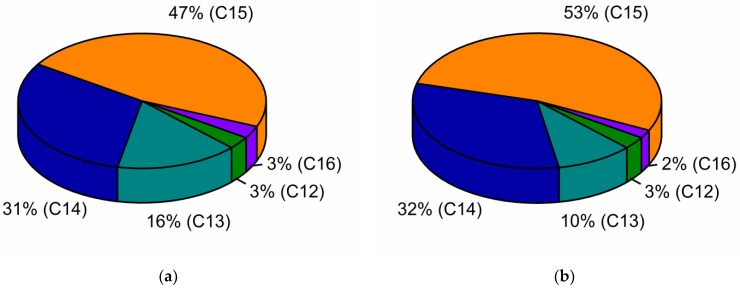
Effect of sunflower cake (**a**) and rapeseed cake (**b**) media on the production of surfactin homologues.

According to the gas chromatography results, the surfactin C_15_ homologue was the dominant fraction in the sunflower (47%) and rapeseed (53%) cake post-culture media (Figure 3). In both media, the concentration of the surfactin C_14_ homologue (31–32%) was also detected. However, significant variability in the concentration of the surfactin C_13_ homologue was noted, reaching 16% in the sunflower and 10% in the rapeseed cake post-fermentation media.

In general, the composition of culture media affects the final surfactin homologue’s profile, in which the profile of FAs is the most important factor. According to de Oliveira Schmidt [34], FAs, as abundant compounds of sunflower and rapeseed cake, act as hydrophobic inducers of biosurfactant production. The oleic and palmitic acid significantly affected the production of surfactin homologues, while the oleic acid induced the greatest diversity of produced homologues. While the concentration of palmitic acid in the sunflower and rapeseed cake was at the same level, 2.82% and 2.44%, significant differences in the oleic acid content (34.96% in sunflower and 77.84% in rapeseed cake) certainly affected the final surfactin homologue profiles’ distinction (Table 1). 

Another factor affecting the hydroxyl FA moiety of surfactin molecules is the amino acid composition in the culture medium [35]. In the media applied in this research, the only sources of amino acids were the hydrolysis products of sunflower and rapeseed cake proteins. Sunflower and rapeseed cake vary in terms of protein concentration (21.4% and 30.2%, respectively) and, most importantly, amino acid composition [36,37]. Hence, as a result of protein hydrolysis, different amino acids were released to the culture media and used in the surfactin molecule biosynthesis process. According to the Hu et al. [35], the presence of Arg, Gln or Val in the culture medium increased the proportion of surfactin homologues with even β-hydroxy FA components (C_14_ and C_16_), whereas the bioavailability of Cys, His, Ile, Leu, Met and Ser enhanced the odd β-hydroxy FAs’ synthesis prevalence.

We demonstrated that variation in the sunflower and rapeseed cake composition affected to a great extent the final proportion of surfactin homologues.

### 2.6. Antioxidant Activity of Surfactin 

Several examples of the use of biosurfactants as antioxidant agents have been previously reported by Nitschke and Silva [38] and Kumar et al. [39]. In another work, the detailed characteristics of surfactin, as an antioxidant agent, were presented. Surfactin, produced in the culture of *B.* *amyloliquefaciens* NS6, showed both ferric-reducing antioxidant power FRAP and DPPH radical scavenging ability. The reducing capacity of surfactin was lower as compared to that of ascorbic acid, but, at the same time, it was around twice as high as in the case of tested rhamnolipids. Moreover, the radical scavenging activity of surfactin was comparable to that presented by a well-known antioxidant, butylated hydroxyanisole (BHA), commonly used as a preservative in food, food packaging, animal feed, cosmetics, rubber and petroleum products [40]. However, the outcomes of our research contradict the results described above. Surfactin produced by *B. subtilis* #309 showed trace antioxidant activity, measured using FRAP, DPPH and ABTS assays (Table 3).

The lack of surfactin activity against DPPH free radicals was demonstrated in another recently published report by Dussert et al. [23]. Here, commercial surfactin, likely produced by *B. subtilis* SD901 (FERM BP-7666) (according to the patent WO2012/043800), was tested at various concentrations (100, 200, 300, 400 and 500 mg/L) for DPPH radical scavenging activity. Eventually, no inhibitory effect was observed. 

The structure of the surfactin molecule could be the reason for such a great difference occurring in the research results of different authors. According to Yalçin and Ҫavuşoǧlu [41], the reduction capacity of lipopeptide biosurfactants, regarding DPPH activity, may be related to the presence of hydroxyl groups in their molecular structure. The chemical structure of surfactin incorporates a common peptide loop of seven amino acids (L-Glu-L-Leu-D-Leu-L-Val-L-Asp-D-Leu-L-Leu), although there exist some homologues that vary in terms of their peptide sequence, such as [Val7] surfactin, [Ile7] surfactin, [Ala4] surfactin, [Asp1, Glu5] surfactin [42]. The number of hydroxyl groups in the surfactin molecule is related to the presence of Glu and Asp, and is constant among all homologues. Moreover, as described in the work of Shao et al. [42], the opening of the lactone ring through the hydrolysis and metylation of the peptide loop chain of surfactin may affect surfactin’s properties. Another aspect that can affect the reducing power of surfactin is the presence of hydrophobic amino acids (Val and Leu) and acidic amino acids (Asp and Glu) in its molecule structure [43]. Surfactin homologue [Ala4], containing alanine in the place of Val, may present reduced activity. Therefore, the final antioxidant activity of surfactin may depend on the structural nuances.

### 2.7. Surfactin as an Angiotensin-Converting Enzyme (ACE)-Inhibitory Agent

ACE is a key component of the rennin-angiotensin system, whose main function is the conversion of angiotensin I to angiotensin II, facilitated by the removal of a dipeptide from the C-terminus of angiotensin I. The role of ACE in mammals is related to the maintenance of stable blood pressure and electrolyte balance. Mammalian ACE activity is typically recognized by measuring the reduction in substrate cleavage in the presence of specific ACE inhibitors such as captopril and lisinopril. Most medical applications involve searching for inhibitors capable of regulating ACE activity in relation to the control of hypertension [44].

Surfactin produced by *B. subtilis* #309 revealed a strong inhibitory effect against ACE activity. The IC_50_ value in the performed studies was 0.62 mg/mL (Table 3). In another work, where enhanced biosurfactant production using isolated *B. mojavensis* I4 was described, a similar effect of the lipopeptide biosurfactant on the ACE activity was detected. A biosurfactant mixture, produced under optimal conditions using glucose and glutamic acid, showed concentration-dependent ACE-inhibitory activity. The highest activity was observed at the concentration of 3 mg/mL in a dose-dependent pattern. The IC_50_ value detected for ACE inhibition was similar to that obtained in our studies and reached 0.7 mg/mL. Moreover, the biosurfactant tested in this research exhibited similar retention times of peaks to those of surfactin, suggesting surfactin as a major biosurfactant in the lipopeptide mixture [45]. 

Biosurfactants’ inhibitory activity was analyzed in vitro as well as in vivo, using an animal model, in the work of Zouari et al. [9]. The lipopeptide mixture used in this study, produced by *B. subtilis* SPB1, was found to comprise surfactin, iturin and fengycin isoforms, as well as two new clusters of lipopeptide isoforms. Lipopeptides, analyzed in vitro, exhibited good ACE inhibition activity (IC_50_ value 1.37 mg/mL) in a dose-dependent pattern. Moreover, the administration of *B. subtilis* SPB1 lipopeptides or atorvastatin in vivo, to hypercaloric high-fat–high-fructose diet (HFFD) rats, decreased significantly the activity of ACE in serum. Notably, the effect of lipopeptide supplementation was better than that induced by atorvastatin, the conventional medication used to treat high cholesterol and limit the risk of stroke, heart attack or other heart complications in patients with type 2 diabetes, coronary heart disease and other risk factors. When lipopeptides were applied throughout 75 days of the experiment, the reduction in ACE activity in serum reached 42%, while the effect of the final 30 days of supplementation was 27.25%, as compared with rats fed HFFD. In comparison, the administration of atorvastatin reduced ACE activity only by 24% [9]. 

The obtained results showed the suitability of surfactin for therapeutic or pharmaceutical purposes as an effective antihypertensive agent.

### 2.8. Molecular Docking of Surfactin C_15_ Homologue to the C- and N-Domains of ACE

The binding affinity of a ligand to the protein-binding site is directly related to the Gibbs energy of binding, which depends on many factors evaluated during molecular docking studies. Due to the properties of the hydrophobic tail, the polar head group and the presence of the donor and acceptor of hydrogen bonds, surfactin-C_15_ could be a potential ACE inhibitor. Therefore, in the present study, we obtained the binding modes of surfactin-C_15_ to the C- and N-domains of ACE. We also analyzed in detail the origins of the stabilization of enzyme-surfactant complexes (Table 4). The results indicated that the origins of stabilization are non-covalent interactions, mainly hydrophobic terms but also hydrogen bonds and van der Waals interactions (with Zn^2+^ ion). 

**Figure 4:**
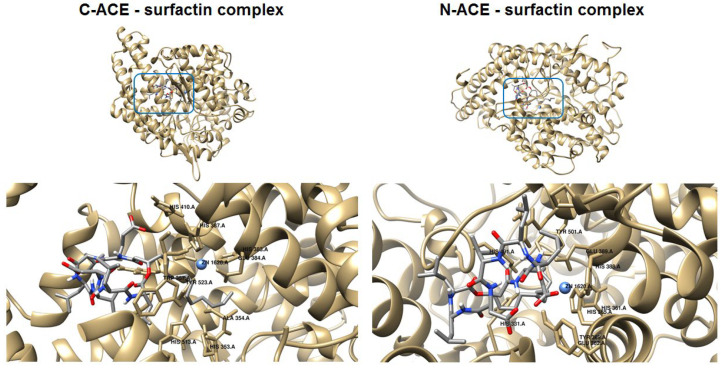
Binding mode of surfactin to C-ACE and N-ACE domains.

In both considered cases, the molecules of the surfactant can bind in close proximity to zinc-binding catalytic sites. The interactions with some amino acid residues that are crucial for the molecular recognition process were identified and are presented in Figure 4. 

**Figure 5:**
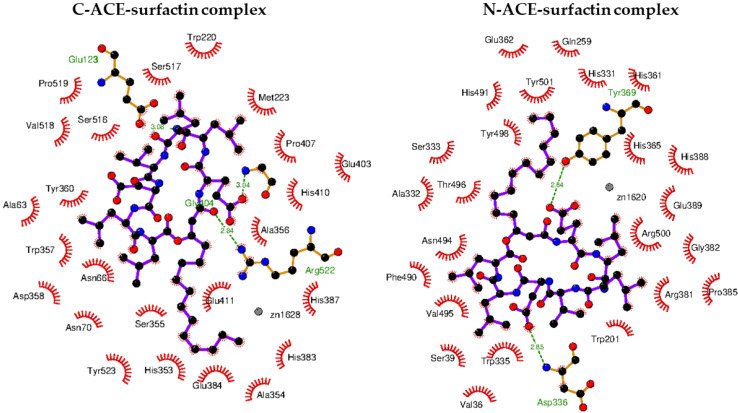
Intermolecular interaction between C-ACE and N-ACE domains with surfactin molecule.

In the case of binding in the C-domain of ACE, surfactin-C_15_ is involved in hydrophobic interactions with His353, Ala354, His382, Glu384 and Tyr523, similar to captopril, which is reported as a catalytic ACE inhibitor. Additionally, the amino acids Ala63, Trp220, Met223, Val518 and Pro519 are localized in close proximity to the ligand, which corresponds to the method of binding previously described for ACE inhibitors [46]. As can be seen in Figure 5, in this case, three hydrogen bonds are formed. One of them involves the H-N group of Arg522 and the oxygen atom of surfactin-C_15_ (2.84 Å). The second hydrogen bond is created between the nitrogen atom of Gly104 and the carboxyl moiety of the lipopeptide. The last hydrogen bond is formed between the oxygen atom of Glu123 and nitrogen of the ligand. According to results, the value of the free energy of binding is equal to −9.5 kcal/mol.

The favorable orientation of the ligand to interact with the zinc-binding catalytic site was also obtained in the case of the N-domain of ACE. This particular orientation is stabilized by hydrogen bonds, hydrophobic forces and van der Waals interactions (Table 4 and Figure 5). The hydrophobic tail of the ligand is located in the cavity formed by His331, Ala322, His491 and Tyr501. The head of the surfactant is surrounded by polar and charged amino acid residues such as Trp201, His365, His388, Asn494, Arg381, Glu389 and Arg500. Carboxyl moieties of surfactin-C_15_ form two hydrogen bonds with the side chain residues of Asp336 and Tyr369 of donor–acceptor distance 2.85 Å and 2.84 Å, respectively. In this case, the free energy of binding is equal to −9.2 kcal/mol.

## 3. Materials and Methods

### 3.1. Oil Seed Cakes and Composition Analysis

The sunflower and rapeseed cake used in this study were obtained after the process of cold pressing of oils as a generous gift from OlejTo, a local Polish company, producing a wide range of oil-based products (Wrocław, Poland). 

The chemical composition of oil seed cakes was determined in the National Laboratory for Feedingstuffs, National Research Institute of Animal Production (Lublin, Poland). Dry matter (DM, (%)), neutral detergent fiber (NDF, (% of DM)), acid detergent fiber (ADF, (% of DM)), acid detergent lignin (ADL, (% of DM)) and ash (% of DM) were estimated using gravimetric methods. The crude protein concentration (% of DM) was determined by a Kjeldahl titration method. Sunflower and rapeseed cakes’ fatty acid compositions (FAs, (% of DM)) were determined using gas chromatography–mass spectrometry (GC-MS). The FAs were esterified at 80 °C for 2 h with a methanol solution of 2.5% (*v*/*v*) sulfuric acid. Subsequently, fatty acid methyl esters (FAMEs) were extracted with n-hexane and analyzed on a GC-MS-QP2010 Plus (Shimadzu, Kyoto, Japan) equipped with a Zebron ZB-FAME capillary column (30 m × 0.25 mm × 0.20 μm; Phenomenex, Torrance, CA, USA). Helium (99.999% purity) was applied as a carrier gas at a constant flow rate of 1 mL min^−1^. According to the program applied, the temperature rose from 100 °C to 210 °C at the rate of 3 °C min^−1^; split ratio 1:20. The injector was maintained at 250 °C and the electron impact ion source was maintained at 220 °C. The FAs were identified by comparing the retention time with the FAMEs of the commercial standard (Supelco 37 Component FAME mix, Sigma-Aldrich, St. Louis, MO, USA).

### 3.2. Surfactin Production Process Specificity in the Culture of B. subtilis #309 Performed on Oil Seed Cake Medium

*B. subtilis* #309, used in this study, was originally isolated from a crude oil sample from a Brazilian oil field [47]. The bacterial strain was stored in the form of glycerol stock (20% *v*/*v*) at −80 °C at the Department of Biotechnology and Food Microbiology, Wrocław University of Environmental and Life Sciences (Wrocław, Poland). In order to perform studies regarding biosurfactant production, *B. subtilis* #309 was cultured in 300 mL Erlenmeyer flasks containing 50 mL of the oil cake medium composed of (g/L): sunflower/rapeseed cake (50), MgSO_4_ (1), KH_2_PO_4_ (0.1), K_2_HPO_4_ (0.13), CaCl_2_ (0.5), FeSO_4_·7H_2_O (0.01). Each flask was inoculated using 1% (*v*/*v*) of *B. subtilis* #309 culture. The bacterial inoculum was grown at 37 °C, 160 rpm for 24 h in the LB medium, composed of (g/L): NaCl (10), tryptone (10), yeast extract (5), pH 7 (A&A Biotechnology, Gdynia, Poland). The surfactin production process was conducted for 168 h at 37 °C with rotary agitation (160 rpm). Every 24 h, one flask was collected, centrifuged at 9500× *g* for 20 min, and the cell-free supernatant was tested for the surfactin concentration (g/L), pH, surface tension reduction (ST, (%)) and emulsification index (E_24_, (%)).

In order to determine the surfactin concentration, 100 µL of culture supernatant was dissolved in 900 µL of methanol (Chempur, Piekary Śląskie, Poland) and analyzed using high-performance liquid chromatography (HPLC, (Shimadzu, Kyoto, Japan)) equipped with a Hypersil GOLD column (5 µm; 4.6 × 150 mm). Samples were injected onto a column in a 10 µL value, and eluted with a flow rate of 0.5 mL/min by a 25-min-long program. As the mobile phase, solvent A (0.1% trifluoroacetic acid) and solvent B (0.1% trifluoroacetic acid in acetonitrile) were run according to the following scheme: (% A:B *v*/*v*): 0 min (50:50), 5 min (20:80), 9 min (10:90), 15 min (0:100), 21 min (0:100), 24 min (50:50) and 25 min (50:50). Detection was performed at λ = 210 nm. The surfactin concentration was calculated according to a calibration curve previously prepared with a surfactin standard (≥98.0%, Sigma-Aldrich, St. Louis, MO, USA). Trifluoroacetic acid (99%) and HPLC-grade acetonitrile (99%) were provided by Sigma-Aldrich Corporation (St. Louis, MO, USA). Surface tension (ST) reduction was determined using du Noüy’s ring technique with the aid of a Krüss K6 tensiometer (Krüss, Hamburg, Germany), as described elsewhere [48]. Before each measurement, the tensiometer was calibrated using ultra-pure water. The surface tension of each sample was measured in triplicate at 25 °C. Emulsification activity, i.e., the ability of the emulsion to retain its structure over a defined time period, was estimated as described previously [49]. Supernatant in the volume of 2 mL was mixed with 2 mL of n-hexadecane in a glass test tube and vortexed for 2 min. The mixture was left to stand for 24 h. Finally, the height of the emulsion layer and total height of the mixtures were measured. Each determination was performed for three independent replications. The stability of the emulsion was calculated according to the following equation:

(1) $$E_{24}\;\left(\%\right)=\frac{\mathrm{height}\;\mathrm{of}\;\mathrm{the}\;\mathrm{emulsion}\;\mathrm{layer}}{\mathrm{total}\;\mathrm{height}\;\mathrm{of}\;\mathrm{the}\;\mathrm{mixtures}}\times 100$$

### 3.3. Proteolytic and Lipolytic Properties of B. subtilis #309

#### 3.3.1. Skim Milk and Tributyrin Agar Plate Assay

Proteolytic and lipolytic properties are important factors affecting substrate utilization and therefore the final surfactin production. The lipolytic activity of *B. subtilis* #309 was examined on tributyrin agar plates (1% of peptone, 0.5% of yeast extract and 1% of tributyrin). Here, 5 μL of bacterial culture (OD_600_ = 1.0) was spotted on an agar plate and incubated for 24 and 96 h at 37 °C. A clear zone around the colonies indicated lipase activity.

The screening for proteolytic activity was conducted using skim milk agar plates. Standardized bacterial culture (OD_600_ = 1.0) was dropped in the volume of 5 µL on the agar plate (2% agar, 10% sterile skim milk (0.5%)). Plates were incubated for 24 and 96 h at 37 °C. A clear zone surrounding bacterial colonies, on a milky background, pointed to proteolytic activity. Uninoculated media (5 µL) spotted both on the tributyrin and skim milk agar plates served as a control. Assays were performed in triplicate.

#### 3.3.2. Proteolytic Enzyme Profile Determination 

Proteolytic enzymes secreted in the rapeseed and sunflower cake medium were analyzed using gelatin zymography. The PageRuler Plus Prestained Protein Ladder (10 to 250 kDa) (Thermo Scientific, Rockford, IL, USA) was applied as a molecular weight standard. Analysis was performed for samples taken at 0 (control), 24 and 96 h of culture and analyzed according to Zhang et al. [50], with our own modifications. Initially, each sample was diluted in the loading buffer (62.5 mM Tris–HCl (pH 6.8), 2% sodium dodecyl sulfate (SDS), 25% glycerol, 0.01% bromophenol blue) in a 1:1 ratio. Then, samples were cooled to a temperature of 0 °C and subjected to SDS-PAGE in 10% Tris–glycine SDS–polyacrylamide gels containing 0.1% gelatin. Electrophoresis was carried out at 150 V, under non-denaturing conditions, with maintenance of a low temperature, preventing excessive digestion of the enzyme migration pathway. Then, gels were washed three times in a 30 min cycle, in Triton solution (2.5% Triton X-100, 50 mM Tris–HCl), and incubated overnight in 50 mM Tris–HCl buffer (pH 7.5), containing 5 mM CaCl_2_ and 150 mM NaCl, at 28 °C. Finally, gels were stained with Coomassie Brilliant Blue R250 (0.1% Coomassie blue, 40% methanol, 10% acetic acid) for 3 h, and then destained (40% methanol, 10% acetic acid) until a clear proteolytic band against a blue background became visible.

### 3.4. Oil Recovery Assay

The applicability of the crude biosurfactant produced by *B. subtilis* #309 in enhanced oil recovery was studied using artificially contaminated sand containing 12.5% (*w*/*w*) crude motor oil. All the experiments were performed in triplicate at room temperature, as described by Gudiña et al. [51]. 

### 3.5. Biosurfactant Recovery and Purification

Biosurfactant production was performed according to the methodology described in Section 3.2. Due to the highest surfactin concentration, the production process was terminated after 120 h in both sunflower and rapeseed cake medium. Post-culture medium was centrifuged (9500× *g* for 20 min at 4 °C) and the supernatant was acidified with 6 M HCl until pH 2.0 to precipitate surfactin. The mixture was kept overnight at 4 °C to facilitate the precipitation process. Afterwards, the mixture was centrifuged at 9500× *g* for 20 min at 4 °C in order to separate the crude surfactin pellet. The pellet was dissolved sequentially in deionized water, and the pH was adjusted to 7.0 using concentrated NaOH [52]. An additional purification of surfactin was conducted using solid-phase extraction (SPE). Crude biosurfactant solution was loaded onto cartridges of the Chromabond C_18_ SPE system (Macherey-Nagel, Düren, Germany) and eluted with acetonitrile gradient (40, 60, 80 and 100% acetonitrile–water (*v*/*v*)). Then, the 80% acetonitrile–water (*v*/*v*) solution (containing surfactin) was concentrated with nitrogen drying. Mass spectrometry of the purified surfactin revealed a purity greater than 99%.

### 3.6. Surfactin Homologue Profile Determination with Gas Chromatography–Mass Spectrometry (GC-MS)

The purified surfactin solution was hydrolyzed with 6 mol HCl at 100 °C for 24 h, as described previously [48]. The FAs were extracted at least three times with diethyl ether. The FAs were esterified with 2.5% (*v*/*v*) sulfuric acid in methanol at 80 °C for 2 h. The fatty acid methyl esters (FAMEs) were extracted with n-hexane and analyzed on a GC-MS-QP2010 Plus (Shimadzu, Kyoto, Japan) equipped with a Zebron ZB-FAME capillary column (30 m × 0.25 mm × 0.20 μm; Phenomenex, Torrance, CA, USA). Helium with 99.999% purity was used as a carrier gas at a constant flow rate of 1 mL min^−1^. The temperature was programmed from 100 °C to 210 °C at the rate of 3 °C min^−1^; split ratio, 1:20. The injector was maintained at 250 °C and the electron impact ion source was maintained at 220 °C. FAMEs were identified and analyzed using the NIST database.

### 3.7. Antioxidant Activity

The surfactin antioxidant activity was measured using ABTS, DPPH and FRAP assays. Each measurement was performed in triplicate. The ABTS assay was conducted according to Re et al. [53]. In the first instance, radical cation (ABTS^•+^) solution was prepared by mixing 7 mM ABTS stock solution (Sigma-Aldrich) with 2.45 mM potassium persulfate solution (Sigma-Aldrich). The mixture was incubated in the dark at room temperature for 12–16 h. Then, the reagent absorbance was adjusted to 0.7 (±0.02) at 734 nm, using distilled water as a blank. The antioxidant activity of the biosurfactant was determined by mixing 10 μL of surfactin (1 mg/mL) with 990 μL of ABTS^•+^ solution. The absorbance was measured after 5 min incubation in the dark, using the Spark multimode microplate leader (Tecan Group Ltd., Männedorf, Switzerland), at 734 nm. The standard curve was prepared for Trolox ethanol solution (Sigma-Aldrich, St. Louis, MO, USA) in the range of 0–2000 μM. The activity of surfactin was expressed as the Trolox equivalent antioxidant capacity (TEAC [µM/g]).

In addition, the antioxidant activity of surfactin, expressed as the ability to scavenge DPPH free radicals, was investigated according to Xu et al. [54]. Initially, 190 μL of DPPH ethanol solution (0.1 mM) (Thermo Scientific, Rockford, IL, USA) was mixed with 10 μL of surfactin solution (1 mg/mL), vortexed and incubated for 30 min in the dark. A corresponding blank was prepared as follows: 10 μL of surfactin was mixed with 190 μL of ethanol solution. The sample was centrifuged (12000 rpm) and the absorbance was measured using the Spark multimode microplate leader (Tecan Group Ltd., Männedorf, Switzerland) at 517 nm. The antioxidant activity of surfactin was expressed as Trolox equivalent (TEAC [µM/g]). The standard curve was prepared in the range of 0–1000 μM.

Finally, the FRAP assay was conducted according to Benzie et al. [55]. The reaction, performed at pH = 3.6, involved the reduction of Fe^3+^ to Fe^2+^ in the presence of 2,4,6-trypyridyl-s-triazine, followed by the formation of a colored complex. Initially, a surfactin sample (50 μL at 1 mg/mL) was mixed with 150 μL of the working solution (1A:1B:10C) prepared as follows: A ((10 mM TPTZ (Sigma Aldrich, St. Louis, MO, USA) in 40 mM HCl), B (20 mM FeCl_3_ × 6H_2_O (Sigma Aldrich)), C (0.3 M acetate buffer pH = 3.6). The mixture was incubated for 10 min at room temperature, and then centrifuged (12,000 rpm), and the absorbance was measured with the Spark multimode microplate reader (Tecan Group Ltd.) at 593 nm. Trolox methanol solution in the range of 0.05–0.5 μM/mL was used to prepare calibration curves.

### 3.8. Angiotensin-Converting Enzyme (ACE)-Inhibitory Activity

The ACE-inhibitory activity assay was performed according to Udenigwe and Aluko [56] and quantified by a regression analysis of ACE-inhibitory activity (%) versus surfactin concentration. The IC_50_ value, i.e., the surfactin concentration (µg/mL) that causes 50% inhibition of ACE activity, was determined. The assay was carried out in triplicate.

### 3.9. Molecular Docking of Surfactin to the C- and N-Domains of ACE

The molecular structure of surfactin was optimized at the PM6 level of theory by using the Gaussian 09 package [57]. Solvent effects were included based on the polarizable continuum model (PCM) [58]. In order to perform molecular docking studies, we applied the AutoDock4.2 program [59]. The structures of the C- and N-domains of human angiotensin I-converting enzyme were taken from the Protein Data Bank (PDB ID: 2XY9 and 2XYD, respectively) [46,60]. The Lamarckian genetic algorithm with local search was employed, with a total of 500 runs for both domains. The calculation included the population of 150 individuals with 27,000 generations and 250,000 energy evaluations.

Free energy of binding (ΔG_binding_), estimated during molecular docking, defines the affinity of the ACE–surfactin complex and can be expressed by the following equation:

ΔG_binding_ = [ΔG_intermolecular_ + ΔG_internal_ + ΔG_tors_] − ΔG_unbound_ (2)

The intermolecular interaction energy (ΔG_intermolecular_) is the sum of dispersion, hydrogen bonding, electrostatics and desolvation energies according to the following expression:

(3) ΔGintermolecular=Wvdw +∑i,j(Aijrij12−Bijrij6) + Whbond∑i,jE(t)(Cijrij12−Dijrij10) + Welec∑i,jqiqje(rij)rij+Wsol∑i,j(SiVi+SjVj)e(−rij22σ2)

The analysis of the obtained results was carried out using the UCSF Chimera System and LIGPLOT v.4.5.3 package [61,62]. 

## 4. Conclusions

Initial studies on the proteolytic and lipolytic activity showed the capability of *B. subtilis* #309 to produce hydrolytic enzymes and therefore to utilize oil cake substrates. The chemical compositions of the sunflower and rapeseed cakes promoted high yields for surfactin production. This was due to the high protein concentration, the source of amino acids for surfactin biosynthesis and the presence of FAs. The conducted research demonstrates the numerous application possibilities of *B. subtilis* #309 surfactin. Depending on the needs, surfactin can be synthesized to reduce ST. It can act as an emulsifier, widely used in the cosmetic industry to stabilize the complex, labile structures of cosmetics, or in the food industry, in order to provide a silky, thick consistency that makes the food more attractive to the consumer and improves the taste. Wide-scale application is possible due to the cost-effective production technology based on agri-food by-products. Therefore, large-scale bioremediation becomes possible. Studies showed *B. subtilis* #309 as an agent of great potential to remove oil-derived pollutants from a contaminated environment. Finally, according to our studies, surfactin can act as an effective inhibitor of ACE. In terms of future directions, our results enable subsequent works aimed at introducing surfactin to the market as a safe, environmentally friendly and successful antihypertensive drug.

## Figures and Tables

**Table 1:** Composition of oil seed cakes.

Component	Sunflower Cake	Rapeseed Cake
Dry Matter (DM), (%)	93.8 ± 0.5	93.1 ± 0.5
Crude Protein, (% of DM)	21.4 ± 0.8	30.2 ± 1.2
Fiber		
Neutral Detergent Fiber (NDF), (% of DM)	34.4 ± 1.7	22.6 ± 1.1
Acid Detergent Fiber (ADF), (% of DM)	29.4 ± 1.5	21.0 ± 1.0
Acid Detergent Lignin (ADL), (% of DM)	9.49 ± 0.9	8.79 ± 0.9
FA, (% of DM)	13.5 ± 0.3	12.9 ± 0.5
FA Composition, (%)		
Palmitic C_16:0_	2.82 ± 0.1	2.44 ± 0.1
Oleic C_18:1_	34.96 ± 0.2	77.84 ± 0.4
Linoleic C_18:2_	55.67 ± 0.6	8.05 ± 0.2
Others	6.55 ± 0.2	11.67 ± 0.1
Ash, (% of DM)	5.85 ± 0.4	5.53 ± 0.3

**Table 2:** Percentages of oil recovered with the biosurfactant produced by B. subtilis #309 in the medium containing 5% sunflower cake or rapeseed cake.

Raw Material	Time (h)	Oil Recovered (%)
Sunflower cake	0	0.8 ± 0.2
	24	14.5 ± 0.6
	48	23.1 ± 1.1
	72	26.8 ± 0.4
	96	30.4 ± 0.1
	120	31.6 ± 0.8
	144	31.0 ± 1.1
	168	30.4 ± 1.0
Rapeseed cake	0	1.2 ± 0.3
	24	22.6 ± 1.2
	48	25.2 ± 0.8
	72	27.1 ± 0.2
	96	31.3 ± 0.2
	120	37.1 ± 0.9
	144	36.2 ± 0.4
	168	33.1 ± 1.2

**Table 3:** The antioxidant and angiotensin-converting enzyme (ACE)-inhibitory activity of surfactin secreted by *B. subtilis* #309.

**Surfactin**	**Antioxidant Activity**		
	ABTS	DPPH	FRAP
TEAC [µM/g]	3.77 ± 0.22	11.67 ± 0.8	0.76 ± 0.096
	**ACE-Inhibitory Activity**		
IC_50_ [mg/mL]	0.62 ± 0.04		

**Table 4:** The origins of stabilization of ACE domain–surfactin C_15_ complexes.

Complex	ΔG_binding_ [kcal/mol]	ΔG_intermolecular_ [kcal/mol]	ΔG_vdw_ ΔG_hbond_ ΔG_desolv_ [kcal/mol]	Electrostatic Energy [kcal/mol]	Hydrogen Bonds	Hydrophobic Interactions
C-ACE–surfactin C_15_	−9.5	−17.0	−17.1	0.1	Gly104 Glu123 Agr522	Ala63 Asn66 Asn70 Gly104 Glu123 Trp220 Met233 His353 Ala354 Ser355 Ala356 Trp357 Asp358 Tyr360 His383 Glu384 His387 Glu403 Pro407 His410 Glu411 Ser516 Ser517 Val518 Pro519 Arg522 Tyr523
N-ACE–surfactin C_15_	−9.2	−16.7	−16.8	0.1	Asp336 Tyr369	Val36 Ser39 Trp201 Gln256 His331 Ala332 Ser333 Trp335 Asp336 His361 Glu362 His365 Tyr369 Arg381 Gly382 Pro385 His388 Glu389 Tyr489 Phe490 His491 Val492 Asn494 Thr496 Arg500 Tyr501

## Data Availability

The data presented in this study are available on request from the corresponding author.
